# Lifting the Burden of Sudden Cardiac Arrest Through International Collaboration: The PARQ Project (EU-COST Action)

**DOI:** 10.1016/j.jacadv.2024.101226

**Published:** 2024-09-11

**Authors:** Natália Oliva-Teles, Enrico Baldi, Bernd W. Böttiger, Jean-Philippe Empana, Martin Jonsson, Giuseppe Ristagno, Jacob Tfelt-Hansen, Hanno L. Tan

**Affiliations:** a Centro de Genética Médica Jacinto Magalhães, Unidade Local de Saúde de Santo António, Porto, Portugal; b UMIB–Unit for Multidisciplinary Research in Biomedicine–ICBAS–Instituto de Ciências Biomédicas Abel Salazar, University of Porto, Porto, Portugal; c ITR–Laboratory for Integrative and Translational Research in Population Health, Porto, Portugal; d Center of Bioethics, Faculty of Medicine, University of Porto, Porto, Portugal; e Division of Cardiology, Fondazione IRCCS Policlinico San Matteo, Pavia, Italy; f Cardiac Arrest and Resuscitation Science Research Team (RESTART), Fondazione IRCCS Policlinico San Matteo, Pavia, Italy; g Medical Faculty and University Hospital of Cologne, Cologne, Germany; h European Resuscitation Council, Niel, Belgium; i INSERM, Université Paris Cité INSERM, Paris, France; j Department of Clinical Science and Education, Södersjukhuset, Center for Resuscitation Science, Karolinska Institutet, Stockholm, Sweden; k Department of Pathophysiology and Transplanatation, University of Milan, Italy; l Fondazione IRCCS Ca' Granda Ospedale Maggiore Policlinico, Milan, Italy; m Section of Genetics, Department of Forensic Medicine, Faculty of Medical Sciences, University of Copenhagen, Denmark; n The Department of Cardiology, The Heart Centre, Copenhagen University Hospital, Rigshospitalet, Copenhagen, Denmark; o Department of Clinical and Experimental Cardiology, Amsterdam University Medical Center, University of Amsterdam Amsterdam, the Netherlands; p Netherlands Heart Institute, Utrecht, the Netherlands

**Keywords:** first response treatment, resuscitation, risk prediction, sudden cardiac arrest

**Figure:**
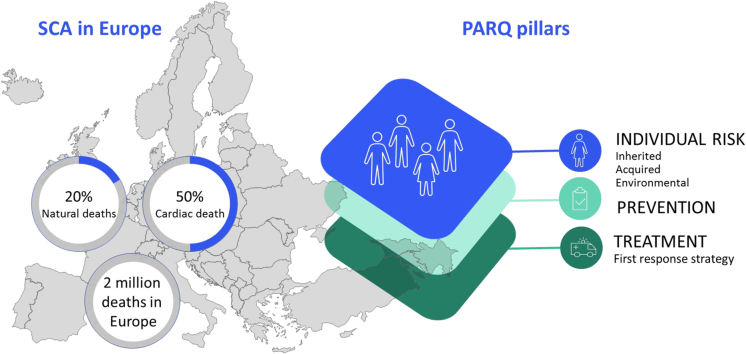

The European Union's COST Action CA19137 “Sudden cardiac arrest prediction and resuscitation network: Improving the quality of care (PARQ)” (2020-2024) has as a primary aim to promote research and knowledge on sudden cardiac arrest (SCA), thereby extending the collaborations and achievements of the PARQ consortium partners, whose core group first came together in the European Union-Horizon 2020 ESCAPE-NET (European Sudden Cardiac Arrest network: towards Prevention, Education, and New Effective Treatment) project of 2017 to 2022. SCA occurs >300,000 times each year in Europe and the United States.^1^ Since SCA strikes unexpectedly and is lethal within minutes if untreated, solving this problem requires: 1) recognizing individuals at risk to design preventive strategies; and 2) providing timely and effective treatment (cardiopulmonary resuscitation). There are large regional and socioeconomic differences in prediction, prevention, risk, and treatment of SCA across Europe.^2^ This translates into different clinical impacts of SCA, eg, its incidence varies, being highest in groups with lower socioeconomic position; similarly, the chances to survive SCA range widely (4%-27%).^2^ The main objective of PARQ was to establish a network of excellence in SCA science that covers all aspects—ranging from clinical and preclinical studies to ethicolegal and socioeconomic studies—which are needed to enable breakthrough developments to better understand the pathobiology of SCA, decrease the incidence of SCA, improve survival rates, and reduce regional and socioeconomic differences therein across Europe (the PARQ pillars) (Figure 1). To cover these required aspects, 4 working groups (WGs) were created: WG1-coordination of sample collection and harmonization of data; WG2-coordination of research aimed at prevention of SCA; WG3-identification of procedural variations in SCA treatment and best practice definition; and WG4-capacity building.

**Figure 1:**
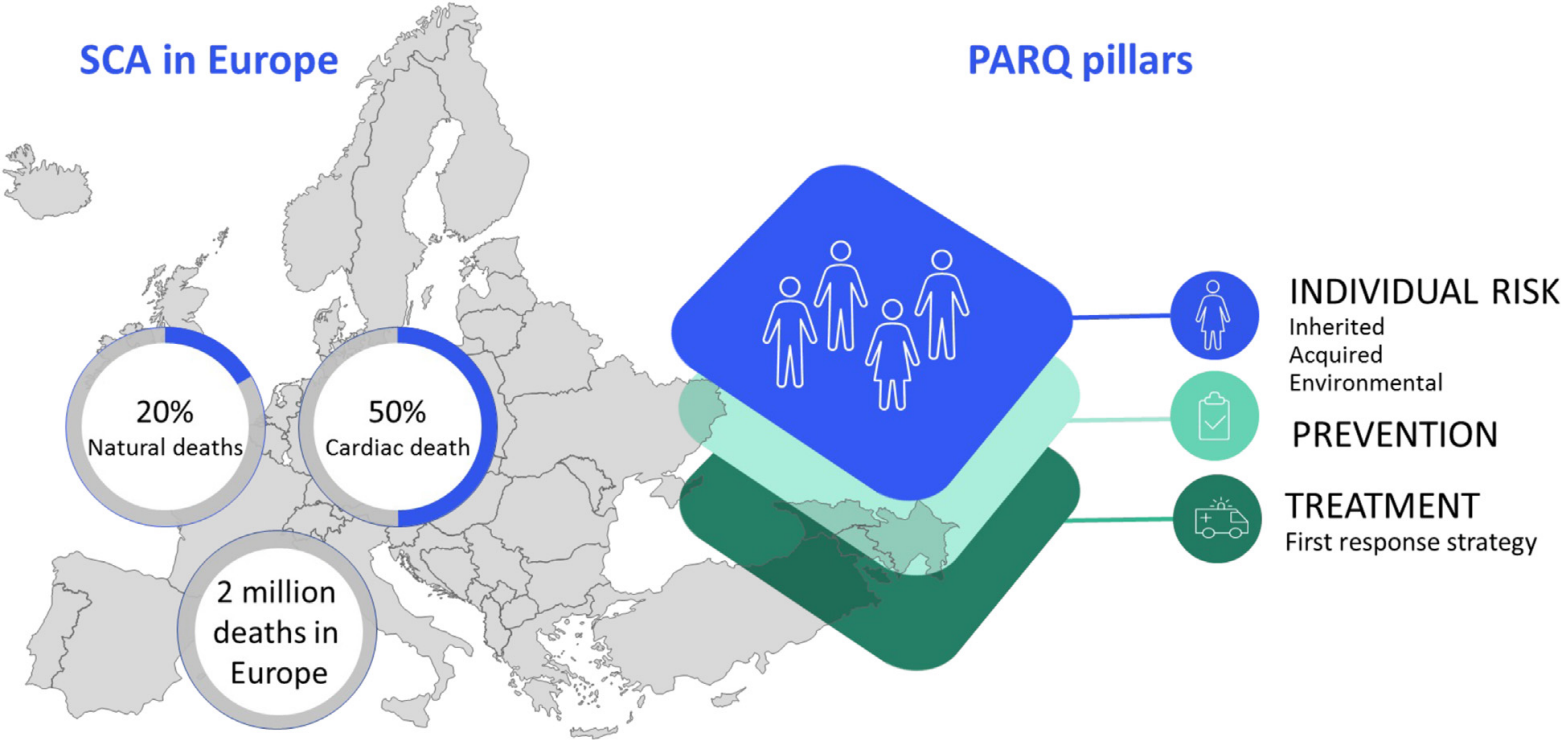
**The PARQ Pillars** “The mission is to share SCA data to develop prevention and treatment guidelines to reduce the overall burden and the differences in survival rates of SCA across europe” (Figure and text from PARQ memorandum of understanding, 2020). SCA = sudden cardiac arrest.

Great success was achieved in working toward the goals as proposed for these WGs, evidenced by the publication of already 57 papers (and counting) across these WGs, including the examples below. WG1 created a proposal for harmonization of sudden cardiac death classification utilizing different methodological approaches. Such harmonization is a crucial basis for SCA research, as it enables comparison of different cohorts with transparent reporting and without loss of information,^3^ thereby laying the groundwork for expansion of the harmonized database of SCA patients created in the ESCAPE-NET project. The harmonization led to the creation of a novel method to assess SCA and sudden cardiac death across different countries. WG1 also included the exploration of the ethicolegal aspects of observational SCA research, and proposed solutions for some dilemmas. Such solutions are required for any research on the sensitive issue of SCA.^4^ WG2 and WG3 utilized the data harmonization efforts of WG1. In WG2, this allowed for a calculation of the incidence of SCA with greater accuracy than previous estimates, based on large SCA cohorts and registries from Paris Sudden Death Expertise Center, Amsterdam Resuscitation Studies, Danish Cardiac Arrest Registry, Swedish Register for Cardiopulmonary Resuscitation, and Lombardia Cardiac Arrest Registry.^1^ Also, validation of the ARIC risk prediction model of SCA became possible,^5^ thus creating a European risk prediction model of SCA and sudden cardiac death. Moreover, an analysis of the impact of the COVID-19 pandemic on the incidence of SCA and on resuscitation strategies was performed;^6^ these insights will increase our preparedness when the next pandemic should strike. In WG3, large variations across Europe in first-responder treatment with associated differences in survival rates were reported.^2^ To facilitate studies aimed at reducing these differences, as a first step, standards to describe first-responder systems (along with smartphone alerting systems, and automated external defibrillator networks) were proposed based on international consensus and expert opinions, with participants from 13 countries, organized under the auspices of the German Resuscitation Council.^7^ Clearly, international collaborations have proven their value, and PARQ has already built a network of SCA researchers among 25 European COST countries (including 10 COST Inclusiveness Target Countries, located mostly in Eastern and Southern Europe), with ongoing inclusion of SCA researchers from other COST countries. In addition, WG4 efforts secured more such collaborations, also with other SCA research groups, eg, the European Resuscitation Council Research NET^8^ and the European Union-Horizon 2020 PROFID (Implementation of personalised risk prediction and prevention of sudden cardiac death after myocardial infarction–European Heart Rhythm Association) project. Crucially, WG4 laid the groundwork to sustain such collaborations well into the future by organizing training schools on various aspects of SCA science, along with junior scientist exchange programs (Short-Term Scientific Missions) that have already yielded various joint publications.^9^

In summary, the PARQ project exemplifies how a strategy of international large-scale scientific collaboration and a holistic research approach is needed and can be successful to lift the worldwide burden of SCA, and to do this in an equitable manner, taking particular care to also serving geographic regions and population groups who now suffer a disproportionally high burden. While PARQ has demonstrably advanced SCA research through collaboration and data harmonization, the established consortium is fully committed to continue this successful strategy well into the future, thereby addressing the key needs in SCA science. In doing so, it continues to identify priority areas that are presently not sufficiently addressed and that will accordingly be added to its research agenda. Examples of such areas are focus on SCA among women^9^ and children,^10^ the role of genetic vulnerability to SCA, and the long-term disabilities and problems that SCA survivors face; developing methods (in a regional context-conscious way) to increase citizen involvement to utilize SCA prevention measures and to provide community first-responder care to SCA victims, and to improve systems of resuscitation care. By focusing on these areas alongside continued collaboration and evaluation of interventions, future initiatives can significantly reduce the global burden of SCA and ensure inclusive and equal opportunity access to improved SCA care and outcome.

## Funding support and author disclosures

This publication is supported by EU-COST Action CA19137 PARQ, financed by 10.13039/501100000921COST (European Cooperation in Science and Technology). Dr Böttiger is treasurer of the European Resuscitation Council (ERC), Founder of the ERC Research NET, Chairman of the German Resuscitation Council (GRC), Member of the “Advanced Life Support (ALS) Task Force of the International Liaison Committee on Resuscitation (ILCOR),” Former Member of the Executive Committee of the German Interdisciplinary Association for Intensive Care and Emergency Medicine (DIVI), Founder of the “Deutsche Stiftung Wiederbelebung,” Federal Medical Advisor of the German Red Cross (DRK), Member of the Advisory Board of the “Deutsche Herzstiftung,” Co-Editor of “Resuscitation,” Editor of the Journal “Notfall + Rettungsmedizin,” Co-Editor of the Brazilian Journal of Anesthesiology. He received fees for lectures from the following companies: Forum für medizinische Fortbildung (FomF), ZOLL Medical Deutschland GmbH, C.R. Bard GmbH, and Becton Dickinson GmbH. All other authors have reported that they have no relationships relevant to the contents of this paper to disclose.
